# Genome Sequence of *Pseudomonas aeruginosa* Strain DK1-NH57388A, a Stable Mucoid Cystic Fibrosis Isolate

**DOI:** 10.1128/genomeA.00008-16

**Published:** 2016-02-25

**Authors:** Anders Norman, Oana Ciofu, Cristina Isabel Amador, Niels Høiby, Lars Jelsbak

**Affiliations:** a Technical University of Denmark, Department of Systems Biology, Kongens Lyngby, Denmark; b Department of Immunology and Microbiology, Costerton Biofilm Center, University of Copenhagen, Copenhagen, Denmark; c Department of Clinical Microbiology, Rigshospitalet, Copenhagen, Denmark

## Abstract

*Pseudomonas aeruginosa* is an important opportunistic pathogen associated with chronic pulmonary infections and mortality in cystic fibrosis (CF) patients. Here, we present the complete genome sequence of stable mucoid *P. aeruginosa* strain DK1-NH57388A, a CF isolate which has previously been used to establish chronic lung infections in an animal model.

## GENOME ANNOUNCEMENT

The Gram-negative bacterium *Pseudomonas aeruginosa* DK1-NH57388A is a stable mucoid isolate, collected in 1997 from the sputum of a Danish cystic fibrosis (CF) patient (CF86) who has had chronic lung infection since 1983. DK1-NH57388A is a representative isolate from the *P. aeruginosa* DK1 lineage which is highly prevalent in Danish CF patients (1, 2). The alginate hyperproducing phenotype of DK1-NH57388A is caused by disruption of the *mucA* anti-sigma factor gene. Furthermore, it has a functional *N*-acyl-homoserine lactone (AHL)-based quorum-sensing (QS) system and produces QS regulated exoproducts including elastase, pyocyanin, and chitinase (3, 4). Due to the stability of the alginate phenotype during several passages, the isolate has been used in an animal model of chronic lung infection (3). This animal model has been used to test the effects of azithromycin, novispirin, cysteamine, oligo(G), and bacteriophages on infection (4–8).

Genomic DK1-NH57388A DNA was isolated using the Wizard genomic DNA purification kit (Promega) according to the manufacturer’s instructions. Genome sequencing was performed on the Illumina MiSeq platform, resulting in 2,797,134 raw 150-bp paired-end reads with a median insert size of 410 bp. Reads were first screened for typical contaminants by using BWA (9) to map against known sequences such as the phiX control phage, leading to the removal of 25,743 reads (0.9%). The SPAdes genome assembler v3.6.2 (10) was then used to *de novo* assemble 417,369,767 quality trimmed (11) bases, resulting in 53 scaffolds (*N*_50_: 351 Kbp, median × coverage: 66.7). A complete draft genome was then constructed in Geneious version R8.3 (12) using the genome sequence of the closely related *P. aeruginosa* DK2 strain (GenBank accession no. CP003149) as a guide. Raw sequencing reads were then mapped back to the draft genome sequence in order to resolve single-nucleotide polymorphisms in repetitive regions and to close gaps in poorly covered regions. The final genome sequence comprises a single circular chromosome, 6,212,531 bp in length, with an average G+C content of 66.6%. Genome size and G+C content are both consistent with other previously sequenced *P. aeruginosa* strains. Automatic genome annotation was performed using Prokka resulting in 5,632 coding regions (CDS), 62 tRNA genes, 12 rRNA genes, and a single transfer-messenger RNA (tmRNA) gene. One of the four rDNA regions contain a disrupted 16S gene due to a 261-bp deletion and a disrupted 23S gene, due to the insertion of the 1,236-bp mobile element IS222. Furthermore, the *mucA* gene has been disrupted by a 105-bp deletion as previously described (3).

The DK1-NH57388A genome is 190 Kbp smaller than the previously described transmissible DK2 strain (6,402,658 bp) which is also prevalent in Danish CF patients (1). The two genomes share 5,988,592 identical sites (90.7%) as revealed by multiple genome alignment using Mauve. Furthermore, DK1 contains two clusters of regularly interspaced short palindromic repeat (CRISPR) systems, one identical to the one found in DK2 (genomic island 5) and one with >95% nucleotide identity to an 11 Kbp CRISPR-cassette found in *P. aeruginosa* RP73 (13). The DK1-NH57388A genome will enable epidemiological studies of the DK1 lineage.

### Nucleotide sequence accession number.

The complete DK1-NH57388A genome sequence has been deposited in the ENA under the accession no. LN870292.
